# The Chemical and Biological Properties of Nanohydroxyapatite Coatings with Antibacterial Nanometals, Obtained in the Electrophoretic Process on the Ti13Zr13Nb Alloy

**DOI:** 10.3390/ijms22063172

**Published:** 2021-03-20

**Authors:** Michał Bartmański, Łukasz Pawłowski, Anna Belcarz, Agata Przekora, Grazyna Ginalska, Gabriel Strugała, Bartłomiej Michał Cieślik, Anna Pałubicka, Andrzej Zieliński

**Affiliations:** 1 Faculty of Mechanical Engineering and Ship Technology, Gdańsk University of Technology, 80-233 Gdansk, Poland; lukasz.pawlowski@pg.edu.pl (Ł.P.); gabriel.strugala@pg.edu.pl (G.S.); andrzej.zielinski@pg.edu.pl (A.Z.); 2 Department of Biochemistry and Biotechnology, Medical University of Lublin, 20-093 Lublin, Poland; anna.belcarz@umlub.pl (A.B.); agata.przekora@umlub.pl (A.P.); grazyna.ginalska@umlub.pl (G.G.); 3 Department of Analytical Chemistry, Faculty of Chemistry, Gdańsk University of Technology, 80-233 Gdansk, Poland; bartlomiej.cieslik@pg.edu.pl; 4 Department of Laboratory Diagnostics and Microbiology with Blood Bank, Specialist Hospital in Kościerzyna, 83-400 Kościerzyna, Poland; apalubicka@op.pl; 5 Department of Oncological Surgery, Medical University of Gdańsk, 80-210 Gdańsk, Poland

**Keywords:** nanometals, nanohydroxyapatite coatings, biocompatibility, antibacterial efficiency, cytotoxicity

## Abstract

The risk of an early inflammation after implantation surgery of titanium implants has caused the development of different antimicrobial measures. The present research is aimed at characterizing the effects of nanosilver and nanocopper dispersed in the nanohydroxyapatite coatings, deposited on the Ti13Zr13Nb alloy, and on the chemical and biological properties of the coatings. The one-stage deposition process was performed by the electrophoretic method at different contents of nanomaterials in suspension. The surface topography of the coatings was examined with scanning electron microscopy. The wettability was expressed as the water contact angle. The corrosion behavior was characterized by the potentiodynamic technique. The release rate of copper and silver into the simulated body fluid was investigated by atomic absorption spectrometry. The antibacterial efficiency was evaluated as the survivability and adhesion of the bacteria and the growth of the biofilm. The cytotoxicity was assessed for osteoblasts. The results demonstrate that silver and copper increase the corrosion resistance and hydrophilicity. Both elements together effectively kill bacteria and inhibit biofilm growth but appear to be toxic for osteoblasts. The obtained results show that the nanohydroxyapatite coatings doped with nanosilver and nanocopper in a one-stage electrophoretic process can be valuable for antibacterial coatings.

## 1. Introduction

The antibacterial or antimicrobial behavior of titanium implants can be achieved by surface modification by the formation of grafts, micro- and nanostructures, and coatings deposited with a variety of methods and containing the most often used nanometals or antibiotics [1]. Recently, antimicrobial peptides [2,3], electrical stimulation of the surface [4,5], antibiotic and nanosilver [6,7], a layer of fluorinated phosphate [8], Zn in the phosphate coating [6], and Au-HAp with a polydopamine layer [9] were proposed as such solutions.

As concerns the nanometals, two are the most popular: either silver/nanosilver [7,10,11,12,13,14,15,16,17,18,19,20,21] and copper/nanocopper [22,23,24]. Their antibacterial efficiency was usually examined by either the colony-forming unit (CFU) index [12,14,22,25,26,27] or inhibition zone [18,23]. The relation between the antibacterial efficacy and nanometal content was different. 

As concerns silver, the antibacterial efficiency approached 99% against *S. aureus* and *E. coli* at 1.5 wt. % [14] and at 6 wt. % of Ag [17]. In the presence of Ag and Zn nanoparticles incorporated into TiO_2_ nanotubular layers, three different bacteria were almost eliminated within three to 24 h of contact [27]. The long-term Ag release was achieved following an appearance of the ionic form into porous Ti [28]. Surprisingly, in reference [18], the antibacterial effects of 0.05% Ag and 0.01% Ag were observed as more profound than that of 0.3% Ag.

For copper, there is a growing trend towards the modification of implants with metal ions exhibiting antibacterial properties, e.g., Zn^2+^, Cu^2+^, and Ag^+^ [29,30,31]. The antibacterial activities of CuHAp [26], La/Cu-HAp [23], and SrCuHAp [22] coatings were demonstrated. The activity of Cu^2+^ ions was limited to the surface of the nanoparticles [25]. The addition of nanoCu into HAp introduced a good activity against *E. coli* and *S. aureus* [24], but it decreased the bioactivity. The thick oxides obtained by plasma electrochemical oxidation (PEO) with a ratio of Ag/Zn up to value 3 destroyed all bacteria in 24 h [32]. However, a similar destroying effect was also reported for Zn alone [33].

Another challenge is introducing the antimicrobial properties without cytotoxic effects to osteoblasts [34]. As concerns silver, a recent study demonstrated that bone cement containing Ag-doped HAp reduced the viability of mouse preosteoblasts [35]. In reference [18], the addition of Ag caused a significant negative impact on cell proliferation the higher the Ag content, the greater the toxicity. On the other hand, no significant difference between the cells on the AgHAp and HAp coatings was reported in reference [36], the Ag incorporated HAp showed excellent osteoblast adhesion [13], and, as stated in reference [12], such coatings did not induce any sort of cytotoxicity.

However, the presence of Cu^2+^ was often cytotoxic to osteoblasts. For Ag- and Cu-doped HAp, a slightly reduced proliferation of human lung fibroblasts was observed [37]. The obtained HAp-Cu nanocomposite coatings exhibited significant cytotoxicity but at 5 wt. % of Cu [38]. The survival ratio of the osteoblasts decreased as the Cu content increased [25]. In other research [39], both Ag and Cu demonstrated considerable cytotoxicity after 24 h. For chitosan modified by Cu ions, at a low content of Cu, it was nontoxic; at a high content of Cu, the coating exerted a cytotoxic effect against mouse fibroblasts [31].

The present research is aimed at the determination of the effects of nanoAg, nanoCu, and both elements together on the properties of the nanoHAp coating. As the substrate, the Ti13Zr13Nb alloy was chosen, because it has mechanical properties much closer to those of the cortical bone [40,41] than for the Ti6Al4V alloy (110 GPa) [42], improving in such a way the biocompatibility and preventing the “shielding effect” and loosening of the implant [43]. Besides, this alloy does not contain Al or V recognized as hazardous for human health [40,44]. The nanoHAp was tested here as the coating material to determine whether nanometals affect different coatings. The electrophoretic deposition (EPD) was chosen among several different methods, as it would be brought out in relatively thin layers well adjacent to the base and possessing better properties [45,46] than the coatings obtained by plasma spraying [47]. The process determinants of electrophoretic deposition were based on the previous research for nanoHAp [48], nanoHAp/nanoAg [20], and nanoHAp/nanoCu coatings [49,50].

As the process determinant, the different nanometallic elements such as nanoAg, nanoCu, or nanoAg+nanoCu were included in the research scheme at the equal content of a single or both elements. The detailed studies of the microstructure and composition of coatings and their biological properties were performed by a variety of techniques. The results were expected to be valuable in assessing the contribution of any or both elements and their synergic effect on the hydrophilicity, corrosion resistance, antibacterial efficiency, and cytotoxicity, all of them essential for further research and implementation as the multifunctional coatings for titanium implants.

## 2. Materials and Methods

### 2.1. Preparation of Specimens

The Ti13Zr13Nb alloy (Xi’an SAITE Metal Materials Development Co., Ltd., Xi’an, China) of the composition shown in Table 1 was used as a substrate. Round samples, 4 mm thick and 15 mm in radius, were cut from the rods. The surface was ground using 220, 500, 800, 1200, and 2000-µm SiC abrasive papers (Struers Company, Krakow, Poland) on a grinding machine (Saphir 330, ATM GmbH, Mammenlzen, Germany) with a rate of 400 rpm. The specimens were cleaned with 2-propanol (99.7%, POCH, Gliwice, Poland) and then with demineralized water (II purity class acc. PN-EN ISO 3696:1999) obtained by a single distillation (HLP 5, HYDROLAB, Straszyn, Poland) in an ultrasonic bath (Sonic-3, Polsonic, Poland) for 60 min in room temperature. After, the samples were immersed in 25% *v*/*v* HNO_3_ for 10 min at room temperature to remove oxides from the surface and finally cleaned again with demineralized water in an ultrasonic bath (Sonic-3, Polsonic, Warsaw, Poland) for 15 min at room temperature.

### 2.2. Electrophoretic Deposition

The EPD was performed in ethanol (99.8%, POCH, Gliwice, Poland) dispersion of powders: nanoHAp, average grain size 20 nm (99% purity, MK Nano, Missisauga, ON, Canada), nanosilver, 30 nm, and nanocopper, 80 nm (both delivered by Hongwu International Group Ltd., Guangdong, China). Four coating types were obtained (Table 2) by adding the appropriate nanopowder(s) to ethanol and mixing it in an ultrasonic bath (Sonic-3, Polsonic, Poland) at room temperature for 1 h. The EPD was performed (power supply MCP/SPN110-01C, Shanghai MCP Corp, Shanghai, China) using the Ti13Zr13Nb alloy sample as a cathode and Pt as an anode at a distance between electrodes of about 10 mm, voltage value 30 V, and deposition time 2 min at room temperature. Afterward, the specimens were dried in ambient air for 24 h at room temperature, then put into a vacuum furnace (PROTHERM PC442, Ankara, Turkey) and heated for 120 min at 800 °C to increase the density of the coatings and the bonding between the coating and Ti13Zr13Nb substrate. The temperature was increased from room temperature at a rate of 200 °C/h. The specimens were cooled with the furnace.

### 2.3. Microstructure of Coatings

The surfaces were examined with the scanning electron microscope (SEM; JEOL JSM-7800 F, Tokyo, Japan).

### 2.4. Corrosion Behavior of nanoHAp Coatings in Simulated Body Fluid

The corrosion tests were made for uncoated, and nanoHAp-coated Ti13Zr13Nb alloy in a potentiodynamic mode using the potentiostat/galvanostat (Atlas 0531, Atlas Sollich, Gdańsk, Poland) in a simulated body fluid (SBF) at 38 °C. The SBF was prepared according to PN-EN ISO 10993-15 and composed of 0.13-g/L (NH_2_)_2_CO, 0.7-g/L NaCl, 1.5-g/L NaHCO_3_, 0.26-g/L Na_2_HPO_4_, 0.2-g/L K_2_HPO_4_, 0.33-g/L KSCN, and 0.5-g/L KCl. The platinum as a counter-electrode and the Ag/AgCl as a reference electrode were used. The polarization started after 5 min, time sufficient to reach the stable open circuit potential. The potentiodynamic tests were made at a potential change rate of 1 mV/s, within a scan range from −600 mV to +1000 mV vs. Ag/AgCl. The corrosion potential (*E_corr_*) and corrosion current density (*i_corr_*) were determined by the Tafel extrapolation method.

### 2.5. Silver Release in Simulated Body Fluid (SBF) Solution

The change in contents of the silver and copper ions were studied in test solutions, following the immersion of nanoHAp/nanoAg/nanoCu specimens in the SBF (prepared according to the procedure applied for the corrosion tests) of the composition shown above for the period ranging between 1 and 28 days at 39 °C. The contents of the Ag^+^ and Cu^2+^ ions were determined with the use of atomic absorption spectrometry (SensAA DUAL, GBC Scientific Equipment Pty Ltd., Hampshire, IL, USA). The wavelengths used for the Ag analysis were 328.10, and Cu was 324.7 nm, and the slit width equaled 0.5 nm in both cases. For the calibrations, the Ag and Cu basic standard solutions of 1000-mg/L contents in 2% HNO_3_ (VWR Chemicals, Radnor, PA, USA) were applied. The standard solutions, at an average concentration of 10 mg/L, were prepared by diluting 1000-mg/L stock solutions. Six standard solutions were made for the calibration curve: 0.1, 0.3, 0.5, 1.0, 2.0, and 2.5 mg/L. The linear regression method was used to determine the calibration curve. The R^2^ coefficient was equal to 0.998, which proved an acceptable linearity.

### 2.6. Wettability Studies

The wettability studies were made using the contact angle goniometer (Attention Theta Life, Biolin Scientific, Espoo, Finland) and a falling water drop technique at room temperature. The tests were repeated three times for each specimen, and the mean values were calculated.

### 2.7. Evaluation of Antibacterial Properties

The samples of nanoHAp and nanoHAp/nanoAg/nanoCu coatings were defatted and cleaned in ethanol (15 min, twice) and placed in 24-well plates (Costar, Corning Inc., Glendale, AZ, USA) individually for each of the antimicrobial tests described below. The plates containing the cleaned samples were sterilized by the ethylene oxide method in a paper/plastic peel pouch (1 h at 55 °C, followed by 20 h of aeration). In the experiments, the reference strain *Staphylococcus epidermidis* ATCC 25923 (from the American Type Culture Collection) was used. The strain was maintained as the stock in sterile microbanks (Technical Service Consultants Limited, Lancashire, UK) at −20 °C. Before use, the bacteria were transferred onto a fresh Mueller-Hinton Agar medium (Biomaxima, Lublin, Poland) and grown for 24 h at 37 °C. Subsequently, the bacteria were transferred into Mueller-Hinton Broth (Biomaxima, Lublin, Poland) and cultured at 37 °C for another 24 h. The suspension was then diluted to the appropriate density directly before the experiment. 

The antibacterial activity of the samples was tested according to the standard JIS Z 2801:2000. The survivability of the bacterial cells was evaluated in triplicate, after 3-h and 24-h incubation at 37 °C, with individual controls for each incubation period. The results were calculated from three experiments as the means ± SD.

In a test on adherence of bacteria, 1 mL of a sterile bacteria suspension (1.0 × 10^8^ cells/mL) in Mueller-Hinton broth, following the McFarland Equivalence Standards and using a PhoenixSpec nephelometer (Becton Dickinson, Franklin Lakes, NJ, USA), was added to each well of the plate containing the tested samples. After incubation of the samples for 1.5 h at 37 °C under stable conditions, the nonadhered bacterial cells were gently washed away using 0.9% NaCl (3 times, 50 mL). *Streptococcus aureus* adhered cells were detected using Biotium dye (Biotium Inc., Fremont, CA, USA), which made it possible to distinguish between live and dead/dying bacterial cells. The visualization and counting of the cells were carried out by fluorescence microscopy (Olympus BX41 microscope equipped with a CC 12 Soft Imaging System camera, Olympus, Tokio, Japan) using CellSens Dimension 1.12 software (Olympus, Tokyo, Japan). The test was performed in duplicate. The results were calculated from ten independently selected areas for each sample as the means ± SD. Afterward, the samples were fixed in 2.5 glutaraldehyde for 24 h, rinsed twice in phosphate-buffered saline (PBS), then dehydrated in a series of ethanol solutions (30–100%) and dried. Fixed surfaces were observed for the bacterial cell presence using the SEM.

### 2.8. Biofilm Formation

Inhibition of the biofilm appearance and bacterial adhesion tests were performed by immersing the specimens in a standardized bacterial suspension that consisted of five clinical isolated bacterial strains: *Staphylococcus aureus*, *Staphylococcus epidermidis*, *Enterococcus faecalis*, *Enterobacter cloacae,* and *Pseudomonas aeruginosa* (supplied by Specialist Hospital in Kościerzyna, Poland). Ten milliliters of each bacterial strain suspension were taken (inoculum—1 × 10^8^ CFU mL^−1^) and added to 50 mL of the liquid medium—Tryptic Soy Bullion (Merck, Warsaw, Poland). The uncoated Ti13Zr13Nb alloy and nanoHAp/nanoAg/nanoCu specimens were sterilized in an autoclave at 120 °C for 30 min. The samples in disc form (20 mm diameter, 2 mm thick) were placed in Eppendorfs; flooded with this bacterial solution (2 mL); and incubated at 37 °C for 7, 14, and 28 days. The SEM observation was used to assess the adhesion of bacteria to the surface.

### 2.9. In Vitro Cytotoxicity Experiments

Cytotoxicity of the samples (uncoated Ti13Zr13Nb alloy and nanoHAp/nanoAg/nanoCu coatings) were tested using the standard human fetal osteoblast cell line (hFOB 1.19) purchased from American Type Culture Collection (ATCC). The cells were maintained in 1:1 Dulbecco’s Modified Eagle Medium (DMEM)/Ham F12 medium (Sigma-Aldrich Chemicals, St. Louis, MO, USA) with the addition of 10% fetal bovine serum (FBS, Pan-Biotech, Aidenbach, Germany) and antibiotics: 300-μg/mL G418, 100-U/mL penicillin, and 100-μg/mL streptomycin (Sigma-Aldrich Chemicals, St. Louis, MO, USA). Osteoblasts were cultured at 34 °C (ATCC recommendations) in a humidified atmosphere of 5% CO_2_ and 95% air. 

The cytotoxicity evaluation was performed according to ISO 10993-5 with the use of fluid extracts of the samples. Extracts were prepared according to ISO 10993-12 via incubation of the tested materials in a complete culture medium for 24 h at 37 °C. The ratio between the material surface area and the volume of the extraction medium was equal to 1.3 cm^2^/mL. The culture medium incubated for 24 h at 37 °C without the tested material served as a negative control of the cytotoxicity. As to assess the osteoblast viability after exposure to the extracts, the cells were seeded in 96-multiwell plates in 100 μL of DMEM/Ham F12 medium at a concentration of 10^5^ cells/mL (104 cells per well). Upon 24-h incubation at 34 °C, the culture medium was discarded and replaced with 100 µL of materials extracts. Osteoblasts were exposed to the extracts for 48 h, and then, their viability was determined using the colorimetric thiazolyl blue tetrazolium bromide (MTT) test (Sigma-Aldrich Chemicals, St. Louis, MO, USA), as described earlier [51,52]. The test was conducted in quadruplicate and repeated in 3 independent experiments (*n* = 3). Osteoblasts viability was evaluated based on the obtained absorbance (Abs) values (measured with a BioTek Synergy H4 Hybrid Microplate Reader, Biotek®, Bad Friedrichshall, Germany) and expressed as a percentage of the Abs obtained with the negative control (showing 100% viability). This test was followed by Tukey’s multiple comparison test (GraphPad Prism 5, v.5.03 Software, San Diego, CA, USA).

Live/dead double-fluorescent staining of cells cultured on the materials was also performed. Before the cell seeding, samples in the form of discs approx. 0.5 mm thick and 8 mm in diameter were placed in a 48-multiwell plate and preincubated for 4 h in the complete culture medium at 34 °C. Osteoblasts were seeded directly on the Ti13Zr13Nb reference specimens and nanoHAp/nanoAg/nanoCu coatings in 500 μL of the medium at a concentration of 5 × 104 cells/sample. Cells grown on polystyrene (PS) in a well of a 48-multiwell plate served as a control. Upon 48-h incubation, hFOB 1.19 osteoblasts were stained with calcein-AM (green fluorescence of viable cells) and propidium iodide (red fluorescence of nuclei of dead cells) using the Live/Dead Double Staining Kit (Sigma-Aldrich Chemicals, St. Louis, MO, USA) following the manufacturers’ protocols. The viability and morphology of the osteoblasts cultured on the samples were analyzed with the use of a confocal laser scanning microscope (Olympus Fluoview equipped with FV1000, Tokyo, Japan).

### 2.10. Statistical Analysis

Statistical analysis of the data was performed using a one-way ANOVA (analysis of variance). The Kolmogorov–Smirnov test was used to assess the normal distribution of the data. Statistical significance was set at *p* < 0.05. All of the results were presented as means ± standard deviation (SD).

## 3. Results

### 3.1. Morphology of Coatings

**Figure 1:**
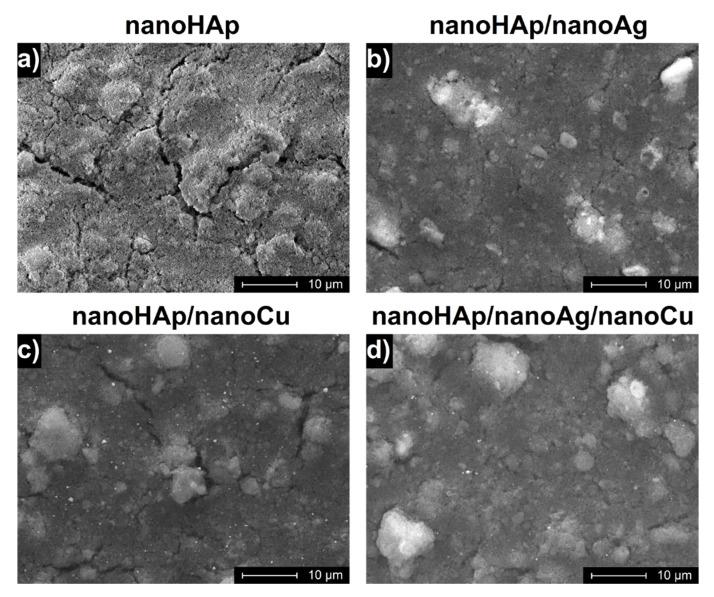
SEM images of the: (**a**) nanoHAp, (**b**) nanoHAp/nanoAg, (**c**) nanoHAp/nanoCu, and (**d**) nanoHAp/nanoAg/nanoCu coatings.

The surface morphologies of the investigated coatings are presented in Figure 1. The shallow cracks occurred, more and longer, especially for the pure nanoHAp coatings. The number and size of the cracks were relatively lower for samples containing nanoAg. Agglomerates of nanoHAp were apparent for all coatings, particularly for the coating with no nanometals addition. The nanoHAp coating showed the highest porosity. In contrary to nanoAg, nanoCu particles were visible and rather uniformly distributed in the obtained nanoHAp coatings.

### 3.2. Corrosion Resistance

**Figure 2:**
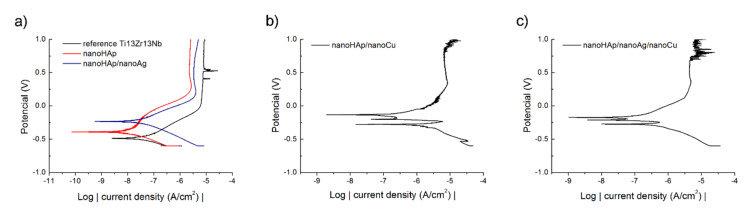
Potentiodynamic curves of the tested: (**a**) reference Ti13Zr13Nb alloy, nanoHAp and nanoHAp/nanoAg coatings, (**b**) nanoHAp/nanoCu coating and (**c**) nanoHAp/nanoAg/nanoCu coating.

The corrosion results are presented in Figure 2 and Table 3. Figure 2 illustrates the potentiodynamic polarization curves. The runs were similar and showed the passivation region. Only for nanoHAp/nanoCu coatings, two cases of short term decreases in their current values on the cathode polarization curves were found. The lowest value of corrosion potential was recorded for the reference sample of the Ti13Zr13Nb alloy. The presence of silver and/or copper nanoparticles resulted in more noble corrosion potentials. A slight increase in the corrosion current density was found for coatings doped with Ag nanoparticles and was significant for coatings containing nanoCu. The highest i_corr_ value was recorded for the nanoHAp/nanoCu sample, indicating the lowest corrosion resistance.

### 3.3. Silver and Copper Release to SBF

The silver and copper releases from the nanoHAp/nanoAg/nanoCu coatings to SBF are presented in Table 4. A lack of detectable silver in the coating and distinct increase in the cumulative concentration of copper in SBF was noticed. The Cu release profile was quite uniform, with no burst release phenomenon. The average Cu concentration increment was 0.033 mg/L per day of exposure, indicating a slow release of Cu from the investigated nanoHAp/nanoAg/nanoCu coating into the SBF solution at 39 °C.

### 3.4. Measurements of the Contact Angle

The results of the water contact angle measurements are presented in Table 5. The averaged contact angle values confirmed the hydrophilic character of the reference Ti13Zr13Nb alloy and all the tested coatings. The average values of the surface contact angle of the nanoHAp coatings both without and with metallic nanoparticles were significantly lower compared to the average value of the contact angle of the titanium alloy substrate. The addition of metallic nanoparticles contributed to the increased wettability of the nanoHAp coatings. With respect to nanoCu, the addition of nanoAg to the nanoHAp coating resulted in a surface with higher wettability. The lowest average contact angle value was recorded for the nanoHAp/nanoAg/nanoCu coating.

### 3.5. Antimicrobial Activity Evaluation and Bacteria Adhesion Evaluation

**Figure 3:**
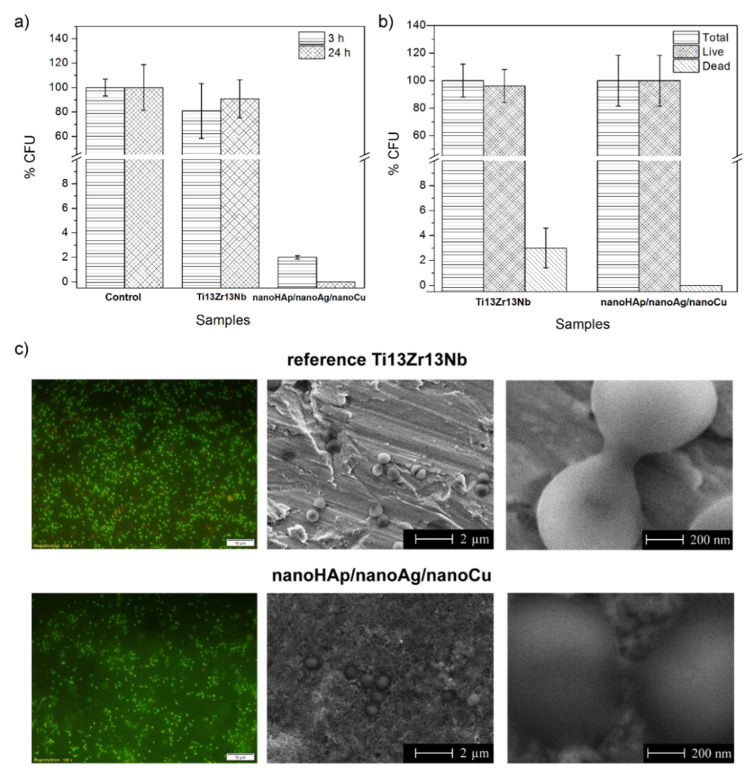
Survival of *S. aureus* cells after incubation of the bacterial cell suspension with tested surfaces for 3 h and 24 h (**a**), amount of *S. aureus* cells adhered to the tested samples after 1.5 h of incubation (**b**), and *S. aureus* cells’ presence on the reference Ti13Zr13Nb alloy and nanoHAp/nanoAg/nanoCu coatings after 1.5 h of contact (fluorescent microscopy—magnification 100× and SEM images with different magnifications) (**c**).

The antibacterial activity of the nanoHAp/nanoAg/nanoCu coatings, estimated according to the JIS Z 2801:2000 standard, was very high. The number of survived planktonic *S. aureus* cells incubated with the surfaces was reduced to 0.2% of the control after three h of incubation and to 0% of the control after 24 h of incubation (Figure 3a).

The results of the *S. aureus* adhesion test showed that the number of bacterial cells adhered to after 1.5 h of contact with the bacterial suspension was lower for the nanoHAp/nanoAg/nanoCu coatings than for the reference Ti13Zr13Nb alloy surfaces (Figure 3b). Unexpectedly, all the bacteria adhered to the nanoHAp/nanoAg/nanoCu coatings were viable. On the contrary, approximately 4% of the cells adhered to the reference surfaces were dead.

The fluorescent microscopy and SEM images of *S. aureus* of the reference Ti13Zr13Nb alloy and nanoHAp/nanoAg/nanoCu coatings at different magnifications are shown in Figure 3c. They confirmed the presence of the adhered bacterial cells on both tested surfaces. The bacteria were mainly of appropriate appearance, both as single and dividing cells with clearly visible division septum, suggesting that the cells were viable.

### 3.6. Inhibition of Biofilm Formation

**Figure 4:**
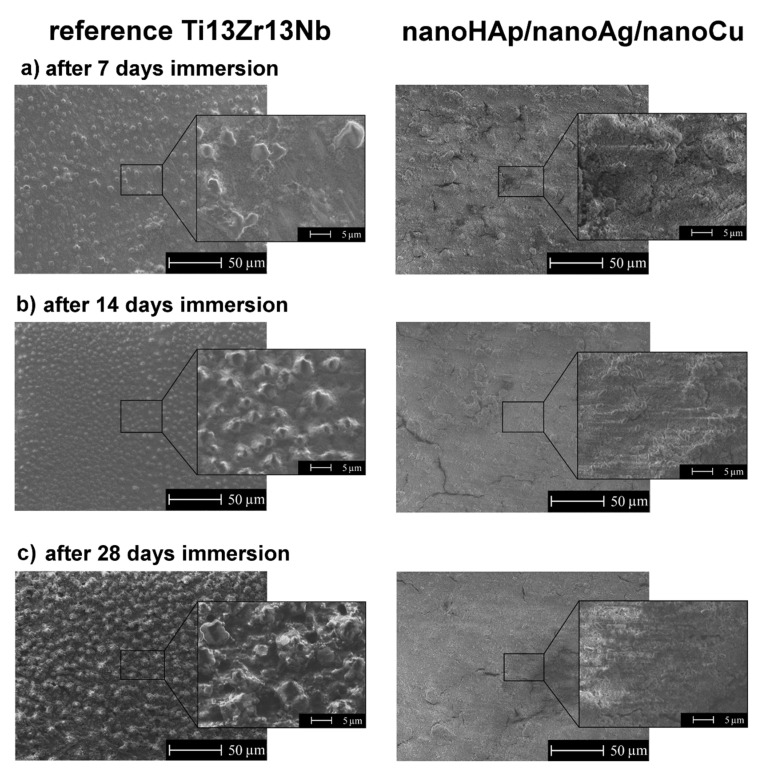
SEM images of the surfaces of reference Ti13Zr13Nb specimens (left column) and nanoHAp/nanoAg/nanoCu coatings (right column) after: (**a**) 7, (**b**) 14 and (**c**) 28 days of exposure in the bacteria solution.

The SEM topography of the reference Ti13Zr13Nb alloy surfaces and nanoHAp/nanoAg/nanoCu coatings after 7, 14, and 28 days of exposure in bacteria’s broth is shown in Figure 4. The remarkable effect of the coating on the retardation of biofilm formation was observed. After 28 days, the biofilm covered the entire surface of the titanium alloy sample, contrary to the surface-modified nanoHAp/nanoAg/nanoCu sample. The deposition of nanoHAp with nanometals significantly reduced the bacterial colonization of the titanium alloy surface.

### 3.7. In Vitro Cell Culture Experiments

**Figure 5:**
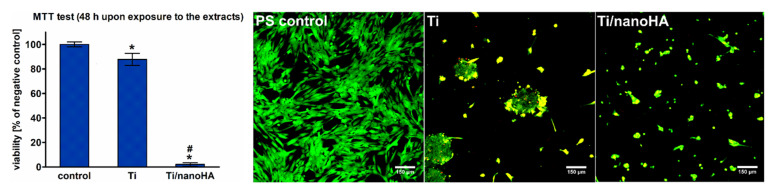
The graph on the left shows cytotoxicity of the materials extracts determined according to the ISO 10993-5 by the MTT test (* significantly different results compared to the negative control of cytotoxicity, # significantly different results compared to the reference Ti13Zr13Nb alloy, according to a one-way ANOVA test followed by Tukey’s multiple comparisons test, *p* < 0.05); Confocal microscope images present live/dead double-fluorescent staining of hFOB 1.19 osteoblasts cultured for 48 h on the materials surfaces (reference Ti13Zr13Nb – image in the middle; nanoHAp/nanoAg/nanoCu coating – image on the right) and polystyrene (PS) well (healthy control cells; image on the left); viable cells—green fluorescence and dead cells—red fluorescence, 100×, scale bar = 150 µm.

An experiment performed according to ISO 10993-5 revealed the high cytotoxicity of the nanoHAp/nanoAg/nanoCu coatings. The viability of hFOB 1.19 cells exposed for 48 h to the extract of the nanoHAp/nanoAg/nanoCu coatings was significantly reduced to 2.1%. In contrast, the viability of the cells exposed to the extract of the reference Ti13Zr13Nb alloy was only slightly decreased to 87.8%, indicating its nontoxicity against eukaryotic cells (according to the ISO 10993-5 material extract, which does not reduce the cell viability by more than 30% and should be considered as nontoxic) (Figure 5).

Live/dead fluorescent staining showed that surfaces of both tested materials (reference Ti13Zr13Nb alloy and nanoHAp/nanoAg/nanoCu coating) were not favorable to osteoblast growth. The reference Ti13Zr13Nb alloy material induced cell aggregation resulting in cell death, whereas the nanoHAp/nanoAg/nanoCu coating was toxic and unsupportive to osteoblast adhesion and proliferation (Figure 5).

## 4. Discussion

The presence of cracks is undoubtedly due to thermal stresses [53] appearing during heating rather than cooling, carried out here at a moderate rate of 200 °C/h. The cracks were relatively short and did not appear in the vast number, so their presence had no substantial effect on the coating adhesion and hardness. The absence of surface cracks was reported for another method, the radio frequency (RF) magnetron sputtering [16], and for drying of the coatings only in the air instead of heating [14].

The presence of nanoparticles of metals during the EPD affected the number and size of the cracks. In each group of nanoHAp coatings, with either nanoAg or nanoCu or both, the reduced number of cracks on the surface of the coating was observed compared to the reference sample. This can be an effect of thermal stress relaxation by the metallic agglomerates. In particular, the number and length of the cracks were the lowest in the nanoHAp/nanoAg/nanoCu coating, presumably by lowering the porosity and increasing the cohesion of coatings in the presence of soft nanometals. The number and length of cracks in the tested coatings can be presumably attributed to the role played by plastic nanometals in brittle ceramics. The improvement of the mechanical properties—in particular, toughness, flexural strength, and resistance to brittle cracking—has been often reported and ascribed to the bridging of cracks by dispersed metal nanoparticles forming ligaments [54]. More recently, the increase in toughness was observed in Ni-containing Al_2_O_3_ composites and explained by an increase in grain boundary strength and decreasing the crack driving energy [55]. In another work [56], the fracture toughness of Mg_2_Si was significantly improved by an addition of Al nanoparticles.

The addition of nanoAg to the nanoHAp coating resulted in a more noble corrosion potential and slightly higher corrosion current density, i.e., decreasing the corrosion resistance, following the previous results [15]. For nanoHAp/nanoCu coatings, even a much higher increase in corrosion current density was observed here. The decreasing corrosion resistance in the presence of nanoparticles of both metals may be attributed to an excellent electrical conductivity of these elements, resulting in a decreasing ohmic contribution to the corrosion resistance of the nanoHAp coating.

The hydrophilicity based on the water drop contact angle values determines the potential biocompatibility in a biological environment, i.e., the anticipated adhesion of molecules on the tested surface. The more hydrophilic the surface, the more organic molecules may be attached to the surface. It is worth mentioning that it is a concurrency in the adhesion of organic molecules, like osteoblasts and bacteria, and high hydrophilicity is a first, but insufficient, condition for the characterization of any surface as able to attach or not to biomolecules. Secondly, the measurements of the contact angle are reliable if the surface is smooth, but, for phosphate coatings, this parameter may be very different. The value of the contact angle is an important parameter describing the wettability or, in other words, for biological environments, the probability of the adhesion of cells on a surface. However, even if the contact angle is low and anticipated adhesion high, only in vivo studies planned for the future will show which of the concurrent cells, e.g., osteoblasts or bacteria, will be preferred. The research described in reference [57] showed that the contact angle values ranging between 1 and 105° did not significantly affect the adhesion of the osteoblasts. On the other side, the adhesion of the bacteria did not correlate with the adhesion of the osteoblasts. The moderately hydrophilic surfaces are the most affecting adhesion of osteoblasts, but the bacteria adhesion is the lowest for superhydrophobic nanocavitated titania surfaces [58]. The effect of the addition of nanometals, however, cannot be described to the wettability of metals that usually reveal high contact angles [59,60,61] but to a different roughness. The wettability method is dedicated to smooth surfaces, and an increasing roughness causes an increase in the contact angle [62,63]. The decreasing contact angle for the tested coatings seems to correlate with decreasing the number of cracks, which, evidently, may affect the roughness and wettability. In this research, all the coatings, as well as the metallic surface, demonstrated hydrophilic properties. However, it is known that adherence of the proteins is lowered on the highly hydrophobic or highly hydrophilic surfaces [64]. Taking this into account, the hydrophilicity for either nanoAg or nanoCu-doped nanoHAp-based coatings is appropriate. Still, if both nanometals are present together in the coating, the contact angle is too low, only about 6°. Similar results, 25–30°, were obtained in reference [12] for AgHAp and 24.1° in reference [22] for CuHAp. On the contrary, in reference [16], the AgHAp coating demonstrated a strong hydrophobicity with a contact angle of 116° or 96°, far beyond the values of the contact angle expected for biocompatible materials. The increasing hydrophobicity was explained by a different grain size rather than different surface chemistry. Despite the last results, the presence of nanometals distinctly increases the hydrophilicity of composite coatings and can be explained by the shallow contact angle of the silver surface, below 10° [65], and high angle for copper, from 60° to even more than 90° [66,67].

The antibacterial studies were made for a single type of coating. Similar studies for Ag or Cu-doped HAp coatings are well-known, but the antibacterial activity has never been examined for the nanoHAp/nanoAg/nanoCu coating. The present results, depending on the applied method and test aims, are divergent. The study of the antibacterial ability of the coatings showed a very high antibacterial efficiency, about 99%. It may be concluded that composite nanoHAp-nanoAg-nanoCu coatings could be successfully applied if only such a test is taken into account.

However, the bacteria adhesion tests showed only a limited positive effect of the tested coatings. The number of bacterial cells adhered to after contact with the bacterial suspension was significantly lower for the nanoHAp/nanoAg/nanoCu coatings than for the reference surfaces. The adhered bacteria were mainly of the appropriate appearance, both as single and dividing cells with clearly visible division septum, suggesting that the cells were viable. This could suggest that the tested coating released some amounts of Ag^+^ and Cu^2+^ ions able to inhibit the adhesion but insufficient to kill them in a short time of 1.5 h. Moreover, the high roughness and wettability of the nanoHAp/nanoAg/nanoCu coatings may promote the adhesion of bacterial cells despite the presence of metallic nanoparticles [68]. The high roughness of the modified coatings also resulted in the nonhomogeneous distribution of adhered bacterial cells on the tested sample. The positive effect, however, appeared and slowly increased during 28-day exposure.

The experiment performed according to the ISO 10993-5 standard revealed a high cytotoxicity of the nanoHAp/nanoAg/nanoCu coatings. That means that the applied contents of the nanometals may be toxic to the eukaryotic cells. The observed cytotoxicity can certainly be attributed to the presence of copper [25,31,37,38,39] rather than to nanosilver, for which such a phenomenon was only occasionally reported [18,35]. Therefore, the medical applications of nanosilver are limited and, of nanocopper, are not observed in clinical practice. Thus, it is challenging to produce an antibacterial and, at the same time, nontoxic implant.

As nanoAg, after a heat treatment, most likely evaporates, its content in the surface becomes very low, but it still affects the biological properties at the interface. The silver evaporation problem is considerable, and the low melting point of the nanometals has already been noted [69,70]. Therefore, no detectable nanoAg content was noticed in the SBF during the release tests, and, for nanoCu, an increase in its concentration in artificial saliva was observed with increasing exposure time. The most substantial increase in the rate of nanoCu release into the test solution was found in the initial period, followed by a slowly decreasing release rate. Compared to previous results, for Ag-substituted hydroxyapatites, in reference [15], the Ag^+^ ions were released quickly over several days, approaching 0.2 ppm on the first day (above 0.1 ppm assumed as the lowest content for the antibacterial effect) and 1.52 ppm on the second day (below the cytotoxic content of 1.6 ppm). A similar gradually increased silver release rate from AgHAp was often observed [12,18]. As concerns the release rate of copper, its total quantity changed from 1.977 ppb to 8.134 ppb per cm^2^ of sample surface [22]. Taking into account the above values, it could be said that silver was present on the surface at a shallow content <0.1 ppm (<100 ppb) and copper at the amount of 7 ppm (700 ppb).

## 5. Conclusions

The electrophoretic deposition of the nanohydroxyapatite, nanosilver, or nanosilver and nanocopper together on the surface of the Ti13Zr13Nb alloy brings out relatively thin composite coatings of the original morphology and biological properties.

The electrophoretic deposition of the composite coatings makes the surface inhomogeneous due to the appearance of numerous aggregates of nanohydroxyapatite and nanometals.

The presence of nanosilver, nanocopper, or both elements together in the composite coating increases the hydrophilicity that seems to have an effect specific to such a ceramic–metallic composite coating.

Nanometals slightly decrease the corrosion resistance of composite coatings, as the metallic elements are more soluble in the simulated body fluid than the hydroxyapatite.

The composite coatings effectively kill the bacteria and prevent a biofilm appearance so that they may be considered active antibacterial compounds.

The composite coatings somewhat demonstrate the cytotoxicity against osteoblasts, apparently due to the presence and biological behavior of nanocopper and copper ions.

To remove two shortcomings, silver evaporation and cytotoxic effects, future research will be performed on the coatings with a distinctly decreased amount of nanocopper, a decreased heat treatment temperature and time, and deposition of nanosilver before and after the heat treatment.

## Figures and Tables

**Table 1:** The chemical composition of the Ti13Nb13Zr alloy, wt. %.

Element	Zr	Nb	Fe	C	N	O	Ti
wt. %	13.0	13.0	0.05	0.04	0.019	0.11	rem.

**Table 2:** Test variables, investigated components, and their contents.

Specimen	Amount of nanoHAp (g/L)	Amount of nanoAg (g/L)	Amount of nanoCu (g/L)
nanoHAp	0.1	-	-
nanoHAp/nanoAg	0.1	0.01	-
nanoHAp/nanoCu	0.1	-	0.01
nanoHAp/nanoAg/nanoCu	0.1	0.005	0.005

**Table 3:** Corrosion parameters of the tested reference Ti13Zr13Nb alloy and nanoHAp-based coatings.

Specimen	E_corr_ (V)	i_corr_ (nA/cm^2^)
reference Ti13Zr13Nb	−0.487	51.92
nanoHAp	−0.379	11.29
nanoHAp/nanoAg	−0.214	32.82
nanoHAp/nanoCu	−0.284	1728.98
nanoHAp/nanoAg/nanoCu	−0.278	1024.01

**Table 4:** Cumulative concentrations of Ag and Cu ions released from the nanoHAp/nanoAg/nanoCu coating after different times of exposure in simulated body fluids (SBF) at 39 °C.

	Concentration (mg/L)	
Days	Ag	Cu
1	<0.100	0.128 ± 0.008
2	<0.100	0.188 ± 0.010
3	<0.100	0.224 ± 0.004
7	<0.100	0.296 ± 0.010
14	<0.100	0.599 ± 0.012
28	<0.100	0.719 ± 0.011

**Table 5:** Water contact angle at room temperature for the tested reference Ti13Zr13Nb alloy and nanoHAp-based coatings (* significantly different results compared to the reference Ti13Zr13Nb alloy, and ^#^ significantly different results compared to the nanoHAp coating, according to a one-way ANOVA test followed by Tukey’s multiple comparison test, *p* < 0.05).

Specimen	Contact Angle (°)
reference Ti13Zr13Nb	53.7 ± 2.1
nanoHAp	35.8 ± 3.5 ^*^
nanoHAp/nanoAg	20.1 ± 2.0 *^,#^
nanoHAp/nanoCu	26.7 ± 2.8 *^,#^
nanoHAp/nanoAg/nanoCu	8.0 ± 1.1 *^,#^

## Data Availability

The data presented in this study are available on request from the corresponding author.

## References

[1] Chouirfa H., Bouloussa H., Migonney V., Falentin-Daudré C. (2019). Review of titanium surface modification techniques and coatings for antibacterial applications. Acta Biomater..

[2] Godoy-Gallardo M., Mas-Moruno C., Fernández-Calderón M.C., Pérez-Giraldo C., Manero J.M., Albericio F., Gil F.J., Rodríguez D. (2014). Covalent immobilization of hLf1–11 peptide on a titanium surface reduces bacterial adhesion and biofilm formation. Acta Biomater..

[3] De Rodríguez López A.L., Lee M.R., Ortiz B.J., Gastfriend B.D., Whitehead R., Lynn D.M., Palecek S.P. (2019). Preventing, *S. aureus* biofilm formation on titanium surfaces by the release of antimicrobial β-peptides from polyelectrolyte multilayers. Acta Biomater..

[4] Ercan B., Kummer K.M., Tarquinio K.M., Webster T.J. (2011). Decreased *Staphylococcus aureus* biofilm growth on anodized nanotubular titanium and the effect of electrical stimulation. Acta Biomater..

[5] Canty M., Luke-Marshall N., Campagnari A., Ehrensberger M. (2017). Cathodic voltage-controlled electrical stimulation of titanium for prevention of methicillin-resistant *Staphylococcus aureus* and *Acinetobacter baumannii* biofilm infections. Acta Biomater..

[6] Su Y., Wang K., Gao J., Yang Y., Qin Y.X., Zheng Y., Zhu D. (2019). Enhanced cytocompatibility and antibacterial property of zinc phosphate coating on biodegradable zinc materials. Acta Biomater..

[7] Croes M., Bakhshandeh S., van Hengel I.A.J., Lietaert K., van Kessel K.P.M., Pouran B., van der Wal B.C.H., Vogely H.C., Van Hecke W., Fluit A.C. (2018). Antibacterial and immunogenic behavior of silver coatings on additively manufactured porous titanium. Acta Biomater..

[8] Joseph Nathanael A., Oyane A., Nakamura M., Mahanti M., Koga K., Shitomi K., Miyaji H. (2018). Rapid and area-specific coating of fluoride-incorporated apatite layers by a laser-assisted biomimetic process for tooth surface functionalization. Acta Biomater..

[9] Xu X., Liu X., Tan L., Cui Z., Yang X., Zhu S., Li Z., Yuan X., Zheng Y., Yeung K.W.K. (2018). Controlled-temperature photothermal and oxidative bacteria killing and acceleration of wound healing by polydopamine-assisted Au-hydroxyapatite nanorods. Acta Biomater..

[10] Zhong Z., Qin J., Ma J. (2015). Electrophoretic deposition of biomimetic zinc substituted hydroxyapatite coatings with chitosan and carbon nanotubes on titanium. Ceram. Int..

[11] Zhang X., Chaimayo W., Yang C., Yao J., Miller B.L., Yates M.Z. (2017). Silver-hydroxyapatite composite coatings with enhanced antimicrobial activities through heat treatment. Surf. Coat. Technol..

[12] Sikder P., Koju N., Ren Y., Goel V.K., Phares T., Lin B., Bhaduri S.B. (2018). Development of single-phase silver-doped antibacterial CDHA coatings on Ti6Al4V with sustained release. Surf. Coat. Technol..

[13] Gokcekaya O., Webster T.J., Ueda K., Narushima T., Ergun C. (2017). In vitro performance of Ag-incorporated hydroxyapatite and its adhesive porous coatings deposited by electrostatic spraying. Mater. Sci. Eng. C.

[14] Yan Y., Zhang X., Li C., Huang Y., Ding Q. (2015). Preparation and characterization of chitosan-silver / hydroxyapatite composite coatings on TiO_2_ nanotube for biomedical applications. Appl. Surf. Sci..

[15] Yan Y., Zhang X., Huang Y., Ding Q., Pang X. (2014). Antibacterial and bioactivity of silver substituted hydroxyapatite/TiO_2_ nanotube composite coatings on titanium. Appl. Surf. Sci..

[16] Grubova I.Y., Surmeneva M.A., Ivanova A.A., Kravchuk K., Prymak O., Epple M., Buck V., Surmenev R.A. (2015). The effect of patterned titanium substrates on the properties of silver-doped hydroxyapatite coatings. Surf. Coat. Technol..

[17] Fu C., Zhang X., Savino K., Gabrys P., Gao Y., Chaimayo W., Miller B.L., Yates M.Z. (2016). Antimicrobial silver-hydroxyapatite composite coatings through two-stage electrochemical synthesis. Surf. Coat. Technol..

[18] Geng Z., Cui Z., Li Z., Zhu S., Liang Y., Liu Y., Li X., He X., Yu X., Wang R. (2016). Strontium incorporation to optimize the antibacterial and biological characteristics of silver-substituted hydroxyapatite coating. Mater. Sci. Eng. C.

[19] Bartmanski M., Cieslik B., Glodowska J., Kalka P., Pawlowski L., Pieper M., Zielinski A. (2017). Electrophoretic deposition (EPD) of nanohydroxyapatite-nanosilver coatings on Ti13Zr13Nb alloy. Ceram. Int..

[20] Bartmanski M. (2017). The Properties of Nanosilver–Doped Nanohydroxyapatite Coating on the Ti13zr13Nb Alloy. Adv. Mater. Sci..

[21] Gokcekaya O., Ueda K., Ogasawara K., Kanetaka H., Narushima T. (2017). In vitro evaluation of Ag-containing calcium phosphates: Effectiveness of Ag-incorporated β-tricalcium phosphate. Mater. Sci. Eng. C.

[22] Huang Y., Hao M., Nian X., Qiao H., Zhang X., Zhang X., Song G., Guo J., Pang X., Zhang H. (2016). Strontium and copper co-substituted hydroxyapatite-based coatings with improved antibacterial activity and cytocompatibility fabricated by electrodeposition. Ceram. Int..

[23] Karthika A. (2018). Aliovalent ions substituted hydroxyapatite coating on titanium for improved medical applications. Mater. Today Proc..

[24] Hidalgo-Robatto B.M., López-Álvarez M., Azevedo A.S., Dorado J., Serra J., Azevedo N.F., González P. (2018). Pulsed laser deposition of copper and zinc doped hydroxyapatite coatings for biomedical applications. Surf. Coat. Technol..

[25] Li Y., Ho J., Ooi C.P. (2010). Antibacterial efficacy and cytotoxicity studies of copper (II) and titanium (IV) substituted hydroxyapatite nanoparticles. Mater. Sci. Eng. C.

[26] Stanić V., Dimitrijević S., Antić-Stanković J., Mitrić M., Jokić B., Plećaš I.B., Raičević S. (2010). Synthesis, characterization and antimicrobial activity of copper and zinc-doped hydroxyapatite nanopowders. Appl. Surf. Sci..

[27] Roguska A., Belcarz A., Zalewska J., Holdyński M., Andrzejczuk M., Pisarek M., Ginalska G. (2018). Metal TiO_2_ Nanotube Layers for the Treatment of Dental Implant Infections. Acs Appl. Mater. Interfaces.

[28] Shivaram A., Bose S., Bandyopadhyay A. (2017). Understanding long-term silver release from surface modified porous titanium implants. Acta Biomater..

[29] Zhu H., Hu C., Zhang F., Feng X., Li J., Liu T., Chen J., Zhang J. (2014). Preparation and antibacterial property of silver-containing mesoporous 58S bioactive glass. Mater. Sci. Eng. C.

[30] Bari A., Bloise N., Fiorilli S., Novajra G., Vallet-Regí M., Bruni G., Torres-Pardo A., González-Calbet J.M., Visai L., Vitale-Brovarone C. (2017). Copper-containing mesoporous bioactive glass nanoparticles as multifunctional agent for bone regeneration. Acta Biomater..

[31] Gritsch L., Lovell C., Goldmann W.H., Boccaccini A.R. (2018). Fabrication and characterization of copper(II)-chitosan complexes as antibiotic-free antibacterial biomaterial. Carbohydr. Polym..

[32] van Hengel I.A.J., Putra N.E., Tierolf M.W.A.M., Minneboo M., Fluit A.C., Fratila-Apachitei L.E., Apachitei I., Zadpoor A.A. (2020). Biofunctionalization of selective laser melted porous titanium using silver and zinc nanoparticles to prevent infections by antibiotic-resistant bacteria. Acta Biomater..

[33] Ye J., Li B., Li M., Zheng Y., Wu S., Han Y. (2020). ROS induced bactericidal activity of amorphous Zn-doped titanium oxide coatings and enhanced osseointegration in bacteria-infected rat tibias. Acta Biomater..

[34] Cross L.M., Thakur A., Jalili N.A., Detamore M., Gaharwar A.K. (2016). Nanoengineered biomaterials for repair and regeneration of orthopedic tissue interfaces. Acta Biomater..

[35] Siek D., Ślósarczyk A., Przekora A., Belcarz A., Zima A., Ginalska G., Czechowska J. (2017). Evaluation of antibacterial activity and cytocompatibility of α-TCP based bone cements with silver-doped hydroxyapatite and CaCO_3_. Ceram. Int..

[36] Molaei A., Yari M., Afshar M.R. (2015). Modification of electrophoretic deposition of chitosan–bioactive glass–hydroxyapatite nanocomposite coatings for orthopedic applications by changing voltage and deposition time. Ceram. Int..

[37] Radovanović Ž., Jokić B., Veljović D., Dimitrijević S., Kojić V., Petrović R., Janaćković D. (2014). Antimicrobial activity and biocompatibility of Ag^+^ - and Cu ^2+^ -doped biphasic hydroxyapatite/α-tricalcium phosphate obtained from hydrothermally synthesized Ag^+^ - and Cu^2+^ -doped hydroxyapatite. Appl. Surf. Sci..

[38] Hadidi M., Bigham A., Saebnoori E., Hassanzadeh-Tabrizi S.A., Rahmati S., Alizadeh Z.M., Nasirian V., Rafienia M. (2017). Electrophoretic-deposited hydroxyapatite-copper nanocomposite as an antibacterial coating for biomedical applications. Surf. Coat. Technol..

[39] Banerjee S., Bagchi B., Bhandary S., Kool A., Amin Hoque N., Thakur P., Das S. (2018). A facile vacuum assisted synthesis of nanoparticle impregnated hydroxyapatite composites having excellent antimicrobial properties and biocompatibility. Ceram. Int..

[40] Abdel-Hady Gepreel M., Niinomi M. (2013). Biocompatibility of Ti-alloys for long-term implantation. J. Mech. Behav. Biomed. Mater..

[41] Jugowiec D., Łukaszczyk A., Cieniek Ł., Kot M., Reczyńska K., Cholewa-Kowalska K., Pamuła E., Moskalewicz T. (2017). Electrophoretic deposition and characterization of composite chitosan-based coatings incorporating bioglass and sol-gel glass particles on the Ti-13Nb-13Zr alloy. Surf. Coat. Technol..

[42] Pylypchuk I.V., Petranovskaya A.L., Gorbyk P.P., Korduban A.M., Markovsky P.E., Ivasishin O.M. (2015). Biomimetic Hydroxyapatite Growth on Functionalized Surfaces of Ti-6Al-4V and Ti-Zr-Nb Alloys. Nanoscale Res. Lett..

[43] Chen Q., Thouas G.A. (2015). Metallic implant biomaterials. Mater. Sci. Eng. R Rep..

[44] Oldani C., Dominguez A. (2012). Titanium as a Biomaterial for Implants. Recent Adv. Arthroplast..

[45] Zhou H., Lee J. (2011). Nanoscale hydroxyapatite particles for bone tissue engineering. Acta Biomater..

[46] Farrokhi-Rad M. (2018). Effect of morphology on the electrophoretic deposition of hydroxyapatite nanoparticles. J. Alloy. Compd..

[47] Harun W.S.W., Asri R.I.M., Alias J., Zulkifli F.H., Kadirgama K., Ghani S.A.C., Shariffuddin J.H.M. (2017). A comprehensive review of hydroxyapatite-based coatings adhesion on metallic biomaterials. Ceram. Int..

[48] Bartmanski M., Zielinski A., Majkowska-Marzec B., Strugala G. (2018). Effects of solution composition and electrophoretic deposition voltage on various properties of nanohydroxyapatite coatings on the Ti13Zr13Nb alloy. Ceram. Int..

[49] Bartmanski M., Zielinski A., Jazdzewska M., Głodowska J., Kalka P. (2019). Effects of electrophoretic deposition times and nanotubular oxide surfaces on properties of the nanohydroxyapatite/nanocopper coating on the Ti13Zr13Nb alloy. Ceram. Int..

[50] Bartmański M., Pawłowski Ł., Strugała G., Mielewczyk-Gryń A., Zieliński A. (2019). Properties of nanohydroxyapatite coatings doped with nanocopper, obtained by electrophoretic deposition on Ti13Zr13Nb alloy. Materials.

[51] Przekora A., Czechowska J., Pijocha D., Sarczyk A., Ginalska G. (2014). Do novel cement-type biomaterials reveal ion reactivity that affects cell viability in vitro?. Cent. Eur. J. Biol..

[52] Kolmas J., Pajor K., Pajchel L., Przekora A., Ginalska G., Oledzka E., Sobczak M. (2017). Fabrication and physicochemical characterization of porous composite microgranules with selenium oxyanions and risedronate sodium for potential applications in bone tumors. Int. J. Nanomed..

[53] Wei M., Ruys A.J., Milthorpe B.K., Sorrell C.C. (2005). Precipitation of hydroxyapatite nanoparticles: Effects of precipitation method on electrophoretic deposition. J. Mater. Sci. Mater. Med..

[54] Rodriguez-Suarez T., Bartolomé J.F., Moya J.S. (2012). Mechanical and tribological properties of ceramic/metal composites: A review of phenomena spanning from the nanometer to the micrometer length scale. J. Eur. Ceram. Soc..

[55] Yıldız B.K., Tür Y.K. (2019). An investigation of equibiaxial flexural strength and hardness properties of Al_2_O_3_–Ni nanocomposites based microstructures with ZrO_2_ and Cr_2_O_3_ additives. Mater. Sci. Eng. A.

[56] Kim G., Lee H., Kim J., Roh J.W., Lyo I., Kim B.W., Lee K.H., Lee W. (2017). Enhanced fracture toughness of Al and Bi co-doped Mg_2_Si by metal nanoparticle decoration. Ceram. Int..

[57] Rahnamaee S.Y., Bagheri R., Vossoughi M., Ahmadi Seyedkhani S., Samadikuchaksaraei A. (2020). Bioinspired multifunctional TiO_2_ hierarchical micro/nanostructures with tunable improved bone cell growth and inhibited bacteria adhesion. Ceram. Int..

[58] Rodriguez-Contreras A., Guadarrama Bello D., Nanci A. (2018). Surface nanoporosity has a greater influence on osteogenic and bacterial cell adhesion than crystallinity and wettability. Appl. Surf. Sci..

[59] Schuster J.M., Schvezov C.E., Rosenberger M.R. (2015). Influence of Experimental Variables on the Measure of Contact Angle in Metals Using the Sessile Drop Method. Procedia Mater. Sci..

[60] Cao L., Luo B., Gao H., Miao M., Wang T., Deng Y. (2021). Structure induced wide range wettability: Controlled surface of micro-nano/nano structured copper films for enhanced interface. J. Mater. Sci. Technol..

[61] Hsueh Y.H., Cheng C.Y., Chien H.W., Huang X.H., Huang C.W., Wu C.H., Chen S.T., Ou S.F. (2020). Synergistic effects of collagen and silver on the deposition characteristics, antibacterial ability, and cytocompatibility of a collagen/silver coating on titanium. J. Alloy. Compd..

[62] Eliaz N., Shmueli S., Shur I., Benayahu D., Aronov D., Rosenman G. (2009). The effect of surface treatment on the surface texture and contact angle of electrochemically deposited hydroxyapatite coating and on its interaction with bone-forming cells. Acta Biomater..

[63] Sarkar S., Roy T., Roy A., Moitra S., Ganguly R., Megaridis C.M. (2021). Revisiting the supplementary relationship of dynamic contact angles measured by sessile-droplet and captive-bubble methods: Role of surface roughness. J. Colloid Interface Sci..

[64] Menzies K.L., Jones L. (2010). The impact of contact angle on the biocompatibility of biomaterials. Optom. Vis. Sci..

[65] Osman M.A., Keller B.A. (1996). Wettability of native silver surfaces. Appl. Surf. Sci..

[66] Orlova E., Feoktistov D., Kuznetsov G. (2015). Investigation of drop dynamic contact angle on copper surface. Epj. Web Conf..

[67] Terpiłowski K., Hołysz L., Rymuszka D., Banach R. (2016). Comparison of contact angle measurement methods of liquids on metal alloys. Ann. Univ. Mariae Curie-SklodowskaSect. Aa Chem..

[68] Wassmann T., Kreis S., Behr M., Buergers R. (2017). The influence of surface texture and wettability on initial bacterial adhesion on titanium and zirconium oxide dental implants. Int. J. Implant. Dent..

[69] Asoro M., Damiano J., Ferreira P. (2009). Size Effects on the Melting Temperature of Silver Nanoparticles: In-Situ TEM Observations. Microsc. Microanal..

[70] Yeshchenko O.A., Dmitruk I.M., Alexeenko A.A., Dmytruk A.M. (2007). Size-dependent melting of spherical copper nanoparticles embedded in a silica matrix. Phys. Rev. B Condens. Matter Mater. Phys..

